# Air Quality and Health Impacts of Future Ethanol Production and Use in São Paulo State, Brazil

**DOI:** 10.3390/ijerph13070695

**Published:** 2016-07-11

**Authors:** Noah Scovronick, Daniela França, Marcelo Alonso, Claudia Almeida, Karla Longo, Saulo Freitas, Bernardo Rudorff, Paul Wilkinson

**Affiliations:** 1 Woodrow Wilson School and Climate Futures Initiative, Princeton University, Princeton, NJ 08544, USA; 2 Department of Social and Environmental Health Research, London School of Hygiene and Tropical Medicine, London WC1E 7HT, UK; Paul.Wilkinson@lshtm.ac.uk; 3 Instituto de Geociências, Universidade Federal do Rio de Janeiro, Rio de Janeiro, RJ 21941-916, Brazil; daniela.franca@igeo.ufrj.br; 4 Faculdade de Meteorologia, Universidade Federal de Pelotas (Federal University of Pelotas), Capão de Leão, RS 35903-087, Brazil; marcelo.alonso@ufpel.edu.br; 5 Instituto Nacional de Pesquisas Espaciais (National Institute For Space Research), São José dos Campos, SP 12227-010, Brazil; almeida@dsr.inpe.br (C.A.); karla.longo@cptec.inpe.br (K.L.); saulo.freitas@cptec.inpe.br (S.F.); 6 Agrosatélite Geotecnologia Aplicada Ltda., Florianópolis, SC 88032-005, Brazil; bernardo@agrosatelite.com.br

**Keywords:** biofuel, ethanol, air quality, emissions, pollution, health, cardiovascular, transport

## Abstract

It is often argued that liquid biofuels are cleaner than fossil fuels, and therefore better for human health, however, the evidence on this issue is still unclear. Brazil’s high uptake of ethanol and role as a major producer makes it the most appropriate case study to assess the merits of different biofuel policies. Accordingly, we modeled the impact on air quality and health of two future fuel scenarios in São Paulo State: a business-as-usual scenario where ethanol production and use proceeds according to government predictions and a counterfactual scenario where ethanol is frozen at 2010 levels and future transport fuel demand is met with gasoline. The population-weighted exposure to fine particulate matter (PM_2.5_) and ozone was 3.0 μg/m^3^ and 0.3 ppb lower, respectively, in 2020 in the scenario emphasizing gasoline compared with the business-as-usual (ethanol) scenario. The lower exposure to both pollutants in the gasoline scenario would result in the population living 1100 additional life-years in the first year, and if sustained, would increase to 40,000 life-years in year 20 and continue to rise. Without additional measures to limit emissions, increasing the use of ethanol in Brazil could lead to higher air pollution-related population health burdens when compared to policy that prioritizes gasoline.

## 1. Introduction

As one of the few alternatives to fossil fuels in the transport sector, liquid biofuels have received increased attention for their potential to help mitigate climate change, improve energy security, and revitalize agricultural economies. Accordingly, use of these plant-based fuels has increased dramatically in recent years; from 2007 to 2014, global ethanol production nearly doubled, and strong growth is expected to continue [1,2,3].

It is also often argued that liquid biofuels are cleaner than fossil fuels, and by implication, better for human health (e.g., [4,5]). The literature on this issue however, is not conclusive. Results from emission experiments show marked sensitivity to the vehicle studied (age and type), operating conditions, blend ratio, and possibly to the biofuel’s feedstock crop [6,7,8,9,10]. There may also be trade-offs between health-relevant pollutants, as some biofuel blends have shown a tendency towards reduced particulate matter (PM) but increased ozone precursors when compared to their fossil fuel counterparts [6,7,8,9]. This variability has led some experts to expect relatively large air quality impacts from moving towards ethanol while others argue that the impacts are likely to be more modest or not meaningful [4,5,7,8,10,11].

Whereas most countries began producing biofuels on an industrial scale relatively recently, the Brazilian biofuel program was initiated in response to the oil crisis of 1973. As a result of this head start, and vast agricultural resources, Brazil is the only country in the world resembling a biofuel (ethanol) society; nearly all new passenger cars in Brazil are “flex-fuel”, capable of using up to 100% ethanol or gasoline, and ethanol meets around a third of the country’s gasoline needs in the transport sector [12,13,14]. Brazil also produces about a quarter of the world’s ethanol, made primarily from sugarcane, with production in 2014/2015 estimated at 28.4 billion liters [1,15].

The Brazilian experience therefore offers the most appropriate case study to assess potential costs and benefits resulting from different biofuel policies. Nevertheless, few studies have explored the air quality and health impacts associated with ethanol production and use in Brazil, despite strong indications of biofuel-related disease burdens; epidemiological studies have linked the pre-harvest burning of sugarcane straw with adverse respiratory outcomes in industry workers and the general population [16,17,18,19], while many Brazilian cities (including São Paulo) have pollution levels far exceeding World Health Organization (WHO) air quality guidelines [20,21].

**Figure 1:**
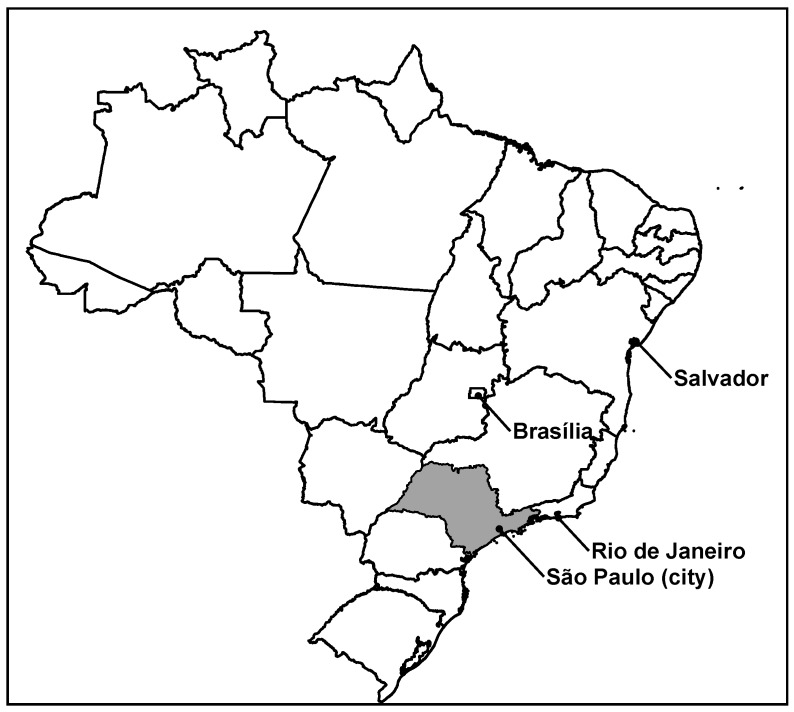
Location of São Paulo State (shaded).

In this paper, we compare the impacts on air quality and health of two future transport fuel scenarios in São Paulo State, Brazil: one where ethanol production and use proceeds according to government predictions and one that instead emphasizes gasoline. São Paulo State (Figure 1) is Brazil’s most populous state (~41.5 million inhabitants), includes its largest metropolitan area (São Paulo), and is responsible for half of the country’s ethanol production [15].

## 2. Materials and Methods

We estimated air quality and associated mortality burdens from exposure to PM_2.5_ and ozone for the 12-month period from October 2019 to September 2020 assuming two different scenarios of future ethanol production and use. October 2019 to September 2020 was chosen instead of a calendar year in order to include one full and continuous warm (October–March) and cold (April–September) season. In both scenarios, and in line with 2007 government projections [22], transport fuel demand in Brazil in 2020 is ~40% higher than 2010 levels, with a total demand of 110,000 MW. Vehicle fleets were also the same in both scenarios, with all new cars assumed to be flex-fuel (see Section 2.1.2 below for details). Where the two scenarios differed was in how the transport energy demand was met, as follows:

Scenario 1: A business-as-usual scenario where ethanol production and use proceed through 2020 according to government projections. In this scenario, fossil fuel demand increases by 46% (12,700 MW) and ethanol by 42% (3800 MW). This scenario is hereafter referred to as the “Ethanol Expansion” scenario.

Scenario 2: A counterfactual “Fossil Fuel” scenario where all additional non-diesel transport energy demand (16,500 MW) after 2010 is met with fossil fuels (gasoline). Ethanol production is therefore frozen at 2010 levels and the only ethanol consumed is either as an additive to gasoline (Brazilian law mandates that gasoline contains ~22% anhydrous ethanol) or in the small number of ethanol-only vehicles still in circulation in 2019/2020 (see Section 2.1.2 below for details). The additional fossil fuel production is assumed to occur outside of the study area.

### 2.1. Emissions

Differences in air quality between the scenarios result from differing levels of two types of emissions: from pre-harvest burning of sugarcane straw and from emissions during end-use of transport fuel.

#### 2.1.1. Emissions from Sugarcane Straw Burning

In certain areas of Brazil, sugarcane fields are burned prior to harvest in order to enable easier access to the cane. The burning season runs between about April and December, a period that approximately corresponds to the cold season. Burning is being phased out in favor of mechanized harvesting, but is expected at least to some degree over the coming years.

To model the associated emissions, we used spatially explicit projections of future sugarcane cultivation and associated burning in São Paulo State (*the* *projections were generated by Dr. Claudia Almeida of Brazil’s National Institute for Space Research (INPE) for a study titled “The spatial scenarios of sugarcane expansion and harvesting practices”. The projections were based on forecasts from the Brazilian Sugarcane Industry Association (UNICA) and data from the CANASAT project coordinated by Dr. Bernardo Rudorff and processed in conjunction with Dr. Daniel Alves Aguiar and Moises Pereira Galvao Salgado (all at INPE). The work is being prepared for publication*). The estimates indicate that the Ethanol Expansion scenario would result in 1,079,207 additional hectares of sugarcane harvested in 2020 compared to the Fossil Fuel scenario and an extra 159,077 hectares burnt (Supplementary Section S1). However, due to the expected increase in coverage of mechanized harvesting, the burnt area declines substantially over time in both scenarios and in 2020 comprises only 5.1% and 3.5% of the total harvest area in the two scenarios, respectively (Supplementary Section S1). Emission factors for sugarcane straw burning are from França et al. [23] and Yokelson et al. [24].

#### 2.1.2. Vehicle Emissions

The vehicle fleet was separated by fuel type and into four vehicle categories: cars, light-duty commercial vehicles, trucks and buses [25]. Circulating vehicle numbers in 2019/2020 were based on licensing data through 2010, a published scrapping rate, and projections of future vehicle sales [25,26,27]. In line with recent trends, all new cars are assumed to be flex-fuel [28] and therefore differences in vehicle emissions result only from the type of fuel consumed; the vehicle fleet and the total transport energy demand is the same in both scenarios (Supplementary Section S2).

Emission factors through 2011 are from data published by São Paulo State’s Environmental agency (CETESB) [29] and we assumed that new vehicles from 2012 onwards had the 2011 emissions factors (Supplementary Section S3). Time-series of projected vehicle emissions (carbon monoxide [CO], oxides of nitrogen (NOx) and volatile organic compounds (VOCs)) through 2020 can be found in Supplementary Section S4. As CETESB does not report primary PM emissions from all vehicle types, we used CO as a proxy and then employed a standard empirical relation between CO and PM_2.5_ to include primary PM_2.5_ emissions in the air quality model [30].

#### 2.1.3. Other Emissions

The only other emission source that was projected forward was from deforestation (slash-and-burn agriculture) unrelated to sugarcane expansion. The future estimates were available from a prior World Bank study [31] and were assumed to be the same in both scenarios. Emissions from other anthropogenic sources and biogenic sources did not change through time in either scenario and were based on emission inventories [32,33,34,35].

### 2.2. Air Quality Modeling

Air quality was modeled with the Coupled Chemistry Aerosol-Tracer Transport model to the Brazilian developments on the Regional Atmospheric Modeling System (CCATT-BRAMS), an on-line regional chemical transport model developed for integrated air quality and weather forecasting and research. It simulates gaseous/aqueous chemistry, transport, dispersion, chemical transformations and removal processes associated with gases and aerosols in the atmosphere and has been described in detail elsewhere [34,36]. Briefly, the model integrates emissions data with meteorological factors and initial and boundary conditions to produce air quality estimates for the modeling domain—in this case São Paulo State. The modeling was conducted at a resolution of 10 km × 10 km and air quality was then determined for every one of the 645 municipal polygons based on the concentration at the location of the municipal seat (municipal polygons were rarely situated entirely within a single grid square). We report annual concentrations and also separated by season (warm and cold).

### 2.3. Health Impact Calculations

We estimated the difference in health that would result from the difference in air quality between the two scenarios using life table methods based on the IOMLIFET model [37,38], populated with age- and sex-specific data for São Paulo State in 2010, as reported by the Brazilian government (and described in Supplementary Section S5) [39,40,41]. Life tables model survival patterns over time, providing estimates of deaths, life-years (LY) lived and life expectancy in each year and for each age-sex group. We allowed a time-horizon (follow-up period) of 106 years, but emphasize impacts over the first 20 years, as the longer 106 year time period would entail strong non-biofuel related influences on health patterns. We assumed that the annual number of births remains constant and that underlying death rates did not change over time.

To model the difference in health between the scenarios, we assumed that age-specific death rates (hazards) in the Ethanol Expansion (business-as-usual) scenario remained constant at 2010 values. Deaths under the counterfactual fossil fuel scenario were computed by applying relative risks to age-specific death rates corresponding to the difference in air pollution concentrations for PM_2.5_ and ozone between the Ethanol Expansion and the counterfactual Fossil Fuel scenario.

Air pollution has been associated with mortality from a wide range of causes [42,43]. We included impacts from three non-overlapping pollutant-outcome pairs, as guided by recent WHO technical reports [43,44], using concentration-response functions from cohort studies of long-term exposure in adults. The first pollutant-outcome pair is cardiovascular mortality from PM_2.5_ exposure and was based on coefficients reported in the meta-analysis by Hoek et al. [45], but updated by Forestiere et al. for the WHO [44]. The meta-analysis includes studies from North America and elsewhere.

The second is PM_2.5_-related mortality from lung cancer quantified with coefficients from Hamra et al. [46]. And the third is ozone-related respiratory mortality from Jerrett et al., [47], which is the only ozone-related outcome that remained significant in two-pollutant models. The concentration-response functions are displayed in Table 1. For both ozone and PM_2.5_ we used (log)linear models without a threshold and did not differentiate between specific components of PM. In line with the study population in the cohort studies, hazards were only applied to adults (30+). As sensitivity analyses, we ran the models using the lower and upper confidence interval of the concentration-response functions (in addition to the central estimates) and with concentration-response functions for all-cause mortality (Table 1).

As we did not model air quality for all years prior to 2019/2020, our modeling approach assumes that the change in air quality occurs instantaneously, as do the corresponding health impacts (no onset lags were included). Therefore, the results should be interpreted as the change in population health that would result from a sustained difference in exposure to ozone and particulate air pollution.

## 3. Results

**Figure 2:**
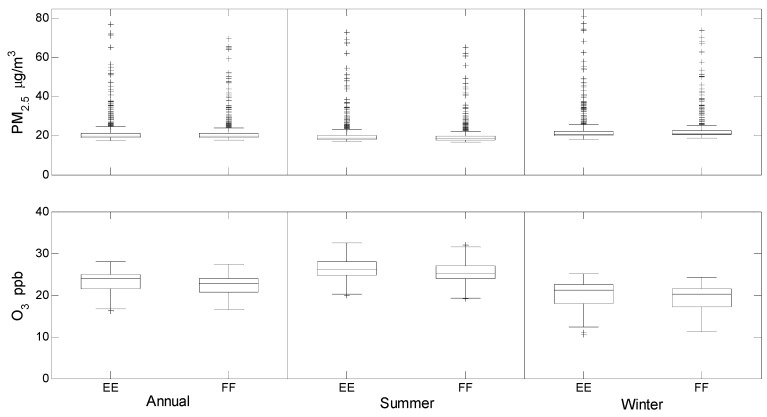
Boxplots summarizing annual, warm and cold season concentrations of fine particulate matter (PM_2.5_) and ozone (O_3_) in the 645 municipalities of São Paulo State in the Ethanol Expansion (EE) and Fossil Fuel (FF) scenarios.

Most of São Paulo State’s 645 municipalities had annual average modeled PM_2.5_ concentrations between 17–25 μg/m^3^, although a small number had much higher concentrations (Figure 2, Supplementary Section S6). The highest concentrations were generally located in urban areas, particularly around São Paulo city, and are in large part attributable to emissions from semi-heavy and heavy-duty diesel trucks, which have been markedly increasing in numbers of late as reflected in both scenarios [29,48]. Higher PM_2.5_ concentrations in the metropolitan region of São Paulo are consistent with recent reporting [29,48,49].

**Figure 3:**
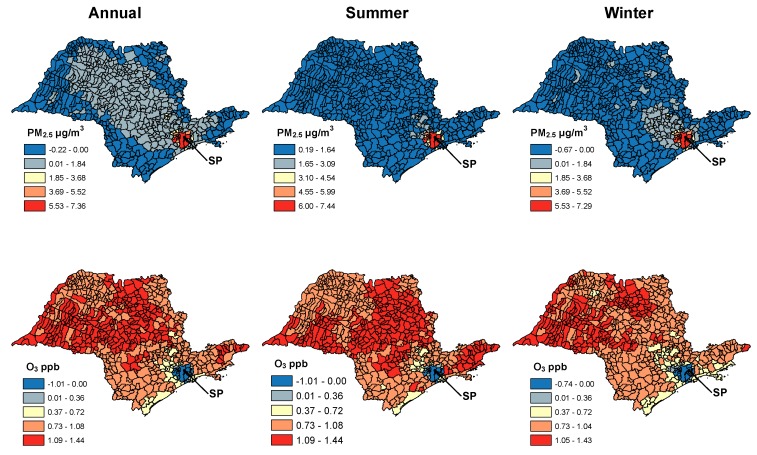
Difference in the concentration of fine particulate matter (PM_2.5_) (average) and ozone (O_3_) (average of 1 h maximums) in each of the 645 municipalities of São Paulo State. Positive values indicate higher concentrations in the Ethanol Expansion scenario.

In 60% of municipalities, the annual average concentration of PM_2.5_ was higher in the Ethanol Expansion compared to the Fossil Fuel scenario, but there was marked seasonal variability: 100% of municipalities were higher in summer in the Ethanol Expansion scenario but only 19% in winter (Figure 3). In the vast majority of municipalities and in all time periods, the difference between the scenarios was ≤1 μg/m^3^ (Figure 3). However, the population-weighted annual average PM_2.5_ concentration in the Ethanol Expansion scenario was 3.0 μg/m^3^ higher than the Fossil Fuel scenario, attributable to the relatively large differences in some highly populated municipalities, including São Paulo municipality which contains 27% of the state’s population (Figure 3, Supplementary Section S7).

Ozone concentrations in both scenarios were mainly between 15–30 ppb (Figure 2, Supplementary Section S6). As with PM_2.5_, ozone concentrations were generally higher in the Ethanol Expansion scenario (Figure 2 and Figure 3), with ~97% of municipalities showing this result in all three time periods. However, of the few municipalities with higher ozone in the Fossil Fuel scenario, many were high-population areas including São Paulo municipality (Figure 3, Supplementary Section S7). The population-weighted warm season ozone concentration in the Ethanol Expansion scenario was 0.29 ppb higher than in the Fossil Fuel scenario.

The lower exposure to both PM_2.5_ and ozone in the Fossil Fuel scenario resulted in more life-years lived by the population of São Paulo State as a whole. The increase was 1100 life-years in the first year, and if sustained, would rise almost linearly to 40,000 in year 20 (Table 2). Gains occurred in all pollutant-outcome pairs, although the vast majority (~90%) was from PM_2.5_-related cardiovascular disease (Table 2, Supplementary Section S8).

**Figure 4:**
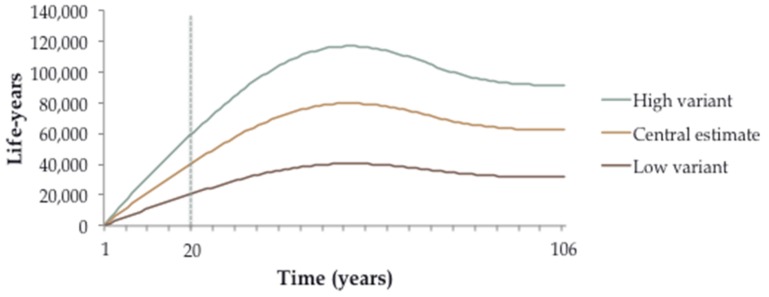
Additional total life-years lived by year in the Fossil Fuel compared to the Ethanol scenario. Low and High variant refers to results estimated using the 5th and 95th confidence intervals in the concentration-response functions, respectively.

Figure 4 projects impacts over the whole 106-year follow-up period, assuming that the differences in air quality are sustained throughout. Benefits continue to accrue to a maximum impact of ~80,000 life-years per year is gained after about 55 years of follow-up. Impacts then decrease slightly to ~60,000 per year and level off. For context, the birth cohort experiencing these reduced risks would have an increased life expectancy of approximately four weeks.

The health impact using concentration-response functions estimated for all-cause mortality is about twice the total of the three specific causes (Supplementary Section S9).

## 4. Discussion

This study suggests that a transport policy promoting ethanol over gasoline would result in more particulate air pollution and higher levels of tropospheric ozone in São Paulo State. As São Paulo State is one of the largest ethanol producing and consuming regions in the world, the results indicate that other countries considering an increase in their use of ethanol should carefully weigh the assumed benefits against potential costs, which we find may include inferior air quality.

Given the estimated difference in pollution (population-weighted differences of 3.0 μg/m^3^ of PM_2.5_ and 0.3 ppb ozone), in the Fossil Fuel scenario the State’s population would live about 1100 additional life-years (50 per million adult population) after one year, and if sustained, this figure would rise to 40,000 life-years (1800 per million adult population) in year 20, all else equal.

Most of the health benefit was associated with exposure to particulate matter and most was from reduced cardiovascular disease. Unsurprisingly, differences were greatest in the high population areas, which are also where most fuel is consumed. The analyses of all-cause mortality suggest that the estimated differences between the scenarios could be conservative, although all-cause mortality is less reliable when applied outside of the population from which the concentration-response functions originated [43]. Had we included potential PM_2.5_ impacts on respiratory diseases in particular, which are often reported in cohort studies, the cause-specific differences between scenarios would have been substantially more pronounced and closer to the estimates based on all-cause mortality; we did not include them because pooled risk coefficients in recent meta-analyses were not (quite) significant at the 5% level [43,44,45].

It is difficult to compare our results with existing studies, as there is substantial variability with respect to the scenarios analyzed, pollutants assessed and the study area, amongst other factors [9]. For example, in a national study of the USA, health costs from corn-based ethanol were higher than gasoline, mainly due to emissions during production, but second-generation ethanol had lower costs [50]. Another study from the USA, which looked specifically at ozone, found that small increases in mortality were likely if vehicles used E85 compared to gasoline [51]. In terms of Brazilian-specific evidence, one study found that adding ethanol to diesel in bus and truck fleet in São Paulo would improve health, but it did not compare gasoline and ethanol or include lighter duty vehicles [52]. More recent research that used highly spatially and temporally resolved observations of road traffic, meteorology and air pollution, together with a consumer demand model found that increased gasoline use in flex-fuel vehicles caused a decline in urban ozone in São Paulo (and an increase in NO and CO), which contrasts with our findings [53]. They did not report results outside of the metropolitan area.

It is important to note that we explored only two of many possible future fuel scenarios—a business-as-usual scenario based on government projections (the “Ethanol Expansion” scenario) and a counterfactual Fossil Fuel scenario. Although the Ethanol Expansion scenario seems the more likely of the two, an important feature of the work is that the Fossil Fuel scenario is also plausible. Brazil’s diverse energy matrix and newly discovered oil reserves, combined with increasing international demand for ethanol, allows it unusual flexibility in designing its domestic energy strategy. Additionally, events from a few years ago demonstrated that ethanol consumption in the country is responsive to factors such as domestic oil prices, which were largely blamed for the reduced domestic ethanol consumption in 2011 and 2012 as compared to previous years (it recovered in 2013 and 2014) [15,54]. Nevertheless, updated Brazilian energy demand projections do not show large differences through 2020 compared to the 2007 report on which this study is based [55].

In addition to the scenario design, the results presented above should be interpreted in light of the assumptions and limitations in the modeling approach, some of which were explored in sensitivity analyses. We quantified expected mortality burdens from multiple causes of death and used concentration-response functions from cohort studies that were mainly conducted in North America and Europe. However, it is not yet clear if those functions are entirely appropriate to the Brazilian population. To account for some of the uncertainty in these parameters, we calculated results using the high and low confidence intervals of the different effect sizes, in addition to the central estimate. The life-years gained using the high variant was approximately three times higher than the low variant, though still not as high as the central estimate when quantifying effects on all-cause mortality, again partly attributable to our exclusion of respiratory causes.

There are a few specific questions about the ozone-mortality association that also require special mention. The first is whether there is a threshold below which mortality does not occur, an issue that is not yet resolved [43]. The municipalities in the study area had ozone concentrations lower than some suggested thresholds for long-term exposure, normally ~35 ppb [43,56]. Therefore, if a threshold for long-term exposure does exist, it is possible that transport fuel choice would not affect ozone-related mortality under these conditions or would affect burdens differently. However, ozone-related effects comprised only a small fraction of the total health impact and did not have a substantive influence on the difference in health impact between the scenarios.

Additionally, there is not much evidence to confirm whether focusing only on summer ozone—normally the case in existing cohort studies [43,47,57]—is appropriate in tropical countries. However, as ozone concentrations were fairly similar in the warm and cold seasons, as were the differences in ozone between the scenarios, results would not be strongly affected by using different seasonal assumptions.

Unlike for the exposure-response parameters, we were not able to test the sensitivity of the air quality estimates to different model parameterizations. This was due to the high computational demands of the CCATT-BRAMS air quality model, which precluded running multiple simulations for each scenario. Three parameters in particular are notable in terms of their potential impact on model results.

The first is the composition of the vehicle fleet, which we assumed was the same in both scenarios. Although it seems almost certain that flex-fuel vehicles will continue to dominate new vehicle sales in Brazil, it is of course not guaranteed, and emission factors do differ depending on whether a vehicle is flex-fuel or conventional gasoline. In 2011, gasoline-only passenger vehicles had lower emissions when compared to flex vehicles using either ethanol or gasoline (see Supplementary Section S3). Light duty commercial vehicles had lower emissions than flex-vehicles driven with ethanol, but flex-gasoline is better in some respects (Supplementary Section S3).

Second, the assumption that all new vehicles have 2011 emissions factors—which was the most recent year available at the time of modeling—does not account for potential changes in vehicle technologies. Although we used emission factors published by São Paulo State’s environmental agency (CETESB), a widely used data source, average vehicle emissions change over time and there is no reason to expect that this would not continue. Nevertheless, more recently published estimates for São Paulo State indicate that new flex-fuel vehicles continue to produce higher emissions of most reported pollutants when using ethanol compared to gasoline (an important exception being NOx in 2013 and 2014, though differences were ≤0.003 g/km) [48,58]. Additionally, the newer data shows that emissions per mile have continued to fall in general, suggesting the absolute levels of emissions estimated here represent an upper limit.

The third is the uncertainty inherent in the projections of future sugarcane burning. The projections we used assume substantial reductions in burning over the coming years. While some will view this assumption as optimistic, many in the industry predict that burning will stop altogether before 2020 [59]. If burning ceased entirely, it would reduce the difference in air quality between the scenarios. However, considering that burning levels were already low in our scenarios and that the meaningful air quality differences occurred mainly in urban areas away from the sugarcane growing regions, it seems unlikely that changes in the burning projections would have a major impact on the study’s conclusions.

Other important limitations in our modeling include the fact that health impacts were estimated for 2019/2020 based on the current (2010) population. Although the population in 2019/2020 will not be substantially different, there will be some changes in terms of its size, age-sex structure and health profile, amongst other factors. Additionally, we made some simplifications about the timing of the air quality and health impacts, which are not entirely realistic. The modeling approach effectively assumes that air quality changes estimated for 2019/2020 would occur suddenly, rather than evolving over time. Similarly, we did not differentiate between health effects that would occur more immediately (e.g., some cardiovascular diseases) and those with longer induction periods (e.g., lung cancer). Therefore, the results presented above represent the health impact that would be expected to arise over the long term if the differences in air quality were sustained, all else equal.

And finally, we only looked at mortality burdens associated with air pollution—and only from ozone and PM_2.5_—when other pathways to health would also be affected by the different fuel scenarios. Examples include possible impacts on occupational health or road traffic injuries if fuel availability (or price) were to affect driving patterns. Much has also been made of the effect of liquid biofuels on food prices, though sugarcane ethanol does not influence food prices to the same extent as some other biofuel feedstock crops [60].

Similarly, our study focused only on mortality, as mortality normally dominates air pollution—related disease burdens [43]. Adding morbidity estimates would be a worthwhile extension of this work considering there is strong evidence of increased hospital admissions from both sugarcane straw burning and urban air pollution in São Paulo State [17,18,61,62]. In general, the impacts we report here should be viewed alongside other potential ancillary benefits or disbenefits of the different fuels.

## 5. Conclusions

Our findings suggest that, contrary to what is often claimed, a transport fuel policy in São Paulo State that prioritizes gasoline over ethanol could result in lower PM_2.5_ and ozone and less premature mortality from air pollution. Specifically, the expansion of ethanol production and use, in line with government projections, could lead to tens of thousands fewer life-years lived per year compared to a fossil fuel scenario. However, we stress that this is not an argument in favor of fossil fuels, but instead demonstrates the need to continue to limit emissions in the transport sector, with strategies likely to include a combination of improved vehicle technologies, economic incentives and modal shifts towards mass transit and active travel.

## Figures and Tables

**Table 1:** Concentration-response functions used in estimating health impact.

Study	Exposure	Mortality Cause *	Percent Change (95% CI)
Main results			
Hoek et al. (2013) [45] and Forestiere et al. (2014) [44]	10 μg/m^3^ PM_2.5_ annual average	Cardiovascular	10.0 (5.0,15.0)
Hamra et al. (2014) [46]	10 μg/m^3^ PM_2.5_ annual average	Lung cancer	9.0 (4.0,14.0)
Jerrett et al. (2009) [47]	10 ppb O_3_ warm-season average of 1 h max	Respiratory ^†^	4.0 (1.3,6.7)
Supplementary analyses			
Hoek et al. (2013) [45] and Forestiere et al. (2014) [44]	10 μg/m^3^ PM_2.5_ annual average	All	6.6 (4.0,9.3)

* ICD-10 codes: Cardiovascular = I00-I99, Respiratory = J00-J99, Lung cancer = C33-C34; ^†^ Two-pollutant model (controlled for PM_2.5_); CI = confidence interval.

**Table 2:** Additional life-years lived per year by the population in the Fossil Fuel compared to the Ethanol Expansion scenario at three time points (Year 1, Year 10 and Year 20).

Follow-up	Cause	Additional Life-Years Lived in the Fossil Fuel Scenario ^†^	Additional Life-Years Per Million Population (30+) ^†,^*
Year 1	Total	1140 (580–1670)	50 (30–80)
	*Cardiovascular (PM_2.5_)*	*1060 (550*–*1540)*	*50 (30*–*70)*
	*Lung cancer (PM_2.5_)*	*70 (30*–*100)*	*3 (1*–*5)*
	*Respiratory (O_3_)*	*20 (10*–*30)*	*1 (0*–*1)*
Year 10	Total	20,620 (10,490–30,190)	950 (480–1390)
	*Cardiovascular (PM_2.5_)*	*19,060 (9820*–*27,790)*	*880 (450*–*1280)*
	*Lung cancer (PM_2.5_)*	*1260 (580*–*1910)*	*60 (30*–*90)*
	*Respiratory (O_3_)*	*290 (100*–*480)*	*10 (0*–*20)*
Year 20	Total	39,800 (20,240–58,310)	1840 (930–2690)
	*Cardiovascular (PM_2.5_)*	*36,750 (18,910*–*53,610)*	*1700 (870*–*2470)*
	*Lung cancer (PM_2.5_)*	*2490 (1140*–*3760)*	*120 (50*–*170)*
	*Respiratory (O_3_)*	*550 (180*–*900)*	*40 (30*–*50)*

^†^ Rounded; * Denominator is the baseline population 30+ in the specified year.

## Supplementary Materials

The following are available online at www.mdpi.com/1660-4601/13/7/695/s1, Section S1: Area Harvested and Burnt, Section S2: Transport Energy Demand, Section S3: Vehicle Emission Factors, Section S4: Vehicle Emissions over Time, Section S5: Development of life Tables, Section S6: Maps of Total PM_2.5_ and Ozone in Each Scenario, Section S7: Differences in Air Pollution in Select Groups of Municipalities, Section S8: Health Impacts by Cause, Section S9: Impacts using coefficients for all-cause mortality (PM_2.5_).

## Abbreviations

The following abbreviations are used in this manuscript:

CCATT-BRAMS	Coupled Chemistry Aerosol and Tracer Transport model to the Brazilian developments on the Regional Atmospheric Modeling System
CI	Confidence interval
EE	Ethanol Expansion (scenario)
FF	Fossil Fuel (scenario)
MW	Megawatt
NOx	oxides of nitrogen
PM_2.5_	Fine particulate matter
VOC	Volatile organic compound
WHO	World Health Organization
