# Supplementary material for: Air Quality and Health Impacts of Future Ethanol Production and Use in São Paulo State, Brazil
Source: Int J Environ Res Public Health. 2016 Jul 11;13(7):695. doi: 10.3390/ijerph13070695 (PMC4962236; doi:10.3390/ijerph13070695)
Supplement: Supplementary file 1 [file ijerph-13-00695-s001.pdf]

Supplementary Materials: Air Quality and Health Impacts of Future Ethanol Production and Use in São Paulo State, Brazil

Noah Scovronick, Daniela França, Marcelo Alonso, Claudia Almeida, Karla Longo, Saulo Freitas, Bernardo Rudorff and Paul Wilkinson

Section S1: Area Harvested and Burnt

The projections of future pre-harvest burning of sugarcane straw found in Supplementary Table S1 were generated by Dr. Claudia Almeida of Brazil's National Institute of Space Research (INPE) for a study titled *"The spatial scenarios of sugarcane expansion and harvesting practices"*. The projections were based on forecasts from the Brazilian Sugarcane Industry Association (UNICA) and data from the CANASAT project coordinated by Dr. Bernardo Rudorff and processed in conjunction with Dr. Daniel Alves Aguiar and Moises Pereira Galvao Salgado (all at INPE). The work is being prepared for publication.

Table S1. Area harvested and burned in São Paulo State in each scenario. 2010 values are empirical, while 2020 is projected. Increasing acreage in the Fossil Fuel scenario is from increased demand for sugar (not ethanol, as ethanol does not increase after 2010).

	**Ethanol Expansion**		**Fossil Fuel**	
	**Harvested (ha)**	**Burned (ha)**	**Harvested (ha)**	**Burned (ha)**
**2010**	4,728,135	2,101,110	4,728,135	2,101,110
**2020**	7,443,854	382,763	6,364,647	223,686

Section S2: Transport Energy Demand

Table S2. Energy consumption (in MW) from different transport fuels in Brazil over time in the Ethanol Expansion (EE) and Fossil Fuel (FF) scenarios. Consumption in São Paulo State is assumed proportional to the size of its vehicle fleet.

	**Gasoline ***		**Ethanol**		**Diesel**	
	EE	FF	EE	FF	EE	FF
2010	27,660	27,660	5263	5263	45,825	45,825
2015	34,254	36,146	7182	5261	53,077	53,077
2020	40,388	44,201	9100	5259	60,329	60,329

* Gasoline in Brazil is composed of ~22% ethanol.

Section S3: Vehicle Emission Factors

Supplementary Table S2 lists emission factors for the different vehicle types in 2011, which were assumed to represent emissions for all new vehicles from 2011 to 2020. Data is from the São Paulo State Environmental Company's (CETESB) emission testing laboratory and readers can refer to the source document for emission factors from previous years [1].

Table S3. Emission factors for new vehicles in São Paulo State, 2011 [1].

		CO	HC	NOx	RCHO
		**g/km**			
Passenger cars	Gasoline *	0.26	0.04	0.03	0.002
	Flex-gasoline	0.28	0.04	0.03	0.001
	Flex-ethanol	0.49	0.09	0.03	0.009
Light-duty commercial vehicles	Gasoline *	0.30	0.03	0.02	0.0018
	Flex-gasoline	0.23	0.04	0.03	0.0015
	Flex-ethanol	0.68	0.09	0.04	0.0090
	Diesel	0.16	0.05	0.61	n/a
		**g/kWh**			
Trucks (all diesel)	Light	0.95	0.13	4.41	0.8
	Medium	0.83	0.13	4.55	0.8
	Heavy	0.81	0.11	4.58	0.7
Buses (all diesel)	Urban	0.85	0.09	4.68	0.8
	Highway	0.62	0.16	4.50	0.6

* Gasoline is 78% gasoline and 22% ethanol by volume; CO = carbon monoxide; HC = hydrocarbons; NOx = oxides of nitrogen; RCHO = aldehydes

Section S4: Vehicle Emissions over Time

Supplementary Figure S1 shows modeled vehicle emissions in Brazil from 2010 to 2020. Despite increasing vehicle numbers, reductions occur in all three pollutants because of the lower emission factors associated with fleet upgrading. For example, based on published government data, there was a 96% reduction in fleet-weighted carbon monoxide (CO) emissions for new Brazilian cars running on gasoline between 1988 and 1998 [1]. It declined another 53% between 1998 and 2008. The corresponding values for oxides of nitrogen (NOx) were 87% and 83%. As mentioned in the main text, fleet upgrading was based on licensing data through 2010, a published scrapping rate, and projections of future vehicle sales [2–4].

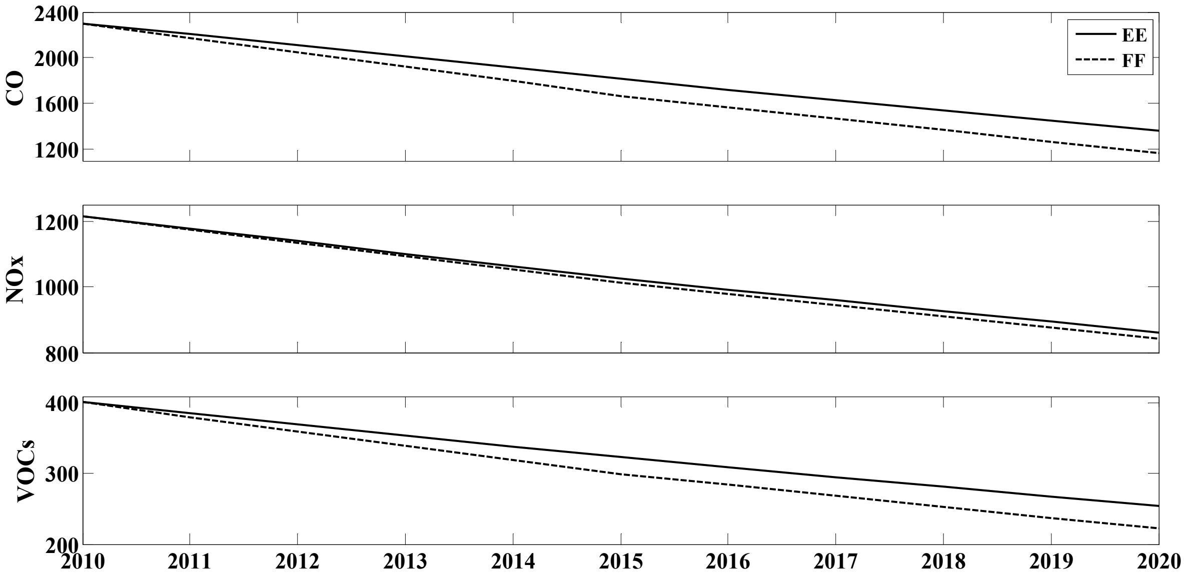

Figure S1. Vehicle emissions (in Gg) of different pollutants over time for the whole of Brazil (emissions in São Paulo State are assumed proportional to the size of the vehicle fleet). EE = Ethanol Expansion scenario. FF = Fossil Fuel scenario.

It is important to note that emissions of $PM_{2.5}$ from vehicles were estimated by the CCATT-BRAMS system based on a relationship commonly used in chemical models to estimate urban PM emissions in terms of the rate associated with emitted CO. The ratios used in the model were provided by the U.S. EPA National Emissions Inventory [5] for all urban anthropogenic sectors over the United States and have a good approximation to South America. The average emission ratio used for $PM_{2.5}$ was 2.95 grams per mole of CO. Therefore, $PM_{2.5}$ emissions in μg/m^2-s from CO emissions in mole/km^2-h were calculated as follows:

$$PM_{2.5}\ (\mu g/m^2\text{-}s) = CO\ (mole/km^2\text{-}h) * 2.95\ (g/mole\ CO) * 106\ (\mu g/g) * 10\text{-}6\ (km^2/m^2)/3600\ (s/h)$$

Section S5: Development of Life Tables

We estimated the difference in health impact between the two scenarios using life table methods based on the IOMLIFET model [6,7], populated with age- and sex-specific population and mortality data for São Paolo State in 2010, as published by the Brazilian government [8–10]. The data was generally reported in five-year age bands and therefore, to populate the year-by-year life tables, we assumed the data was uniformly distributed within the five-year age groups, for both population and mortality.

There was a slight modification for the oldest age group, reported as those people 80 years and older. This group is quite large, particularly in terms of total deaths, and less suited to the assumption of uniformity. For example, it would be unusual to have the same number of deaths in those aged 105 and those aged 80. As a simple adjustment, we used data for all of Brazil that reported population in five-year age bands all the way up to 100 (as mentioned, São Paulo data only went to 80) and determined the percentage of the 80+ population that fell into those five-year categories. We then used the percentages to modify the São Paulo data (population and mortality) accordingly, again assuming uniformity within the five-year age groups.

Section S6: Maps of Total $PM_{2.5}$ and Ozone in Each Scenario

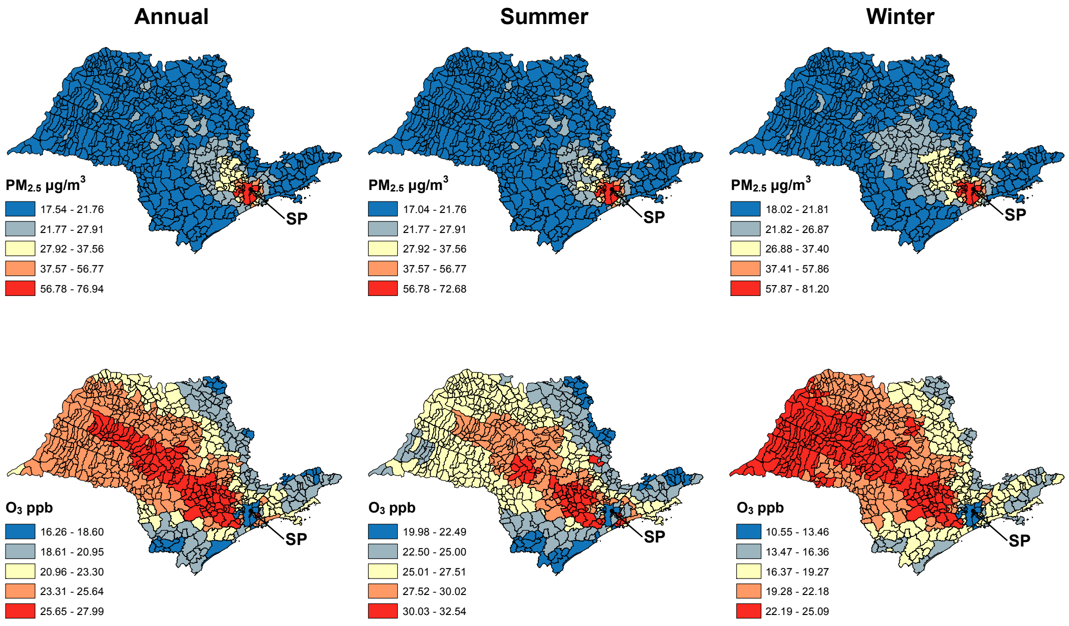

Figure S2. Fine particulate matter ($PM_{2.5}$) (annual average) and ozone (O_3) (average of 1 h maximums) in the Ethanol Expansion scenario.

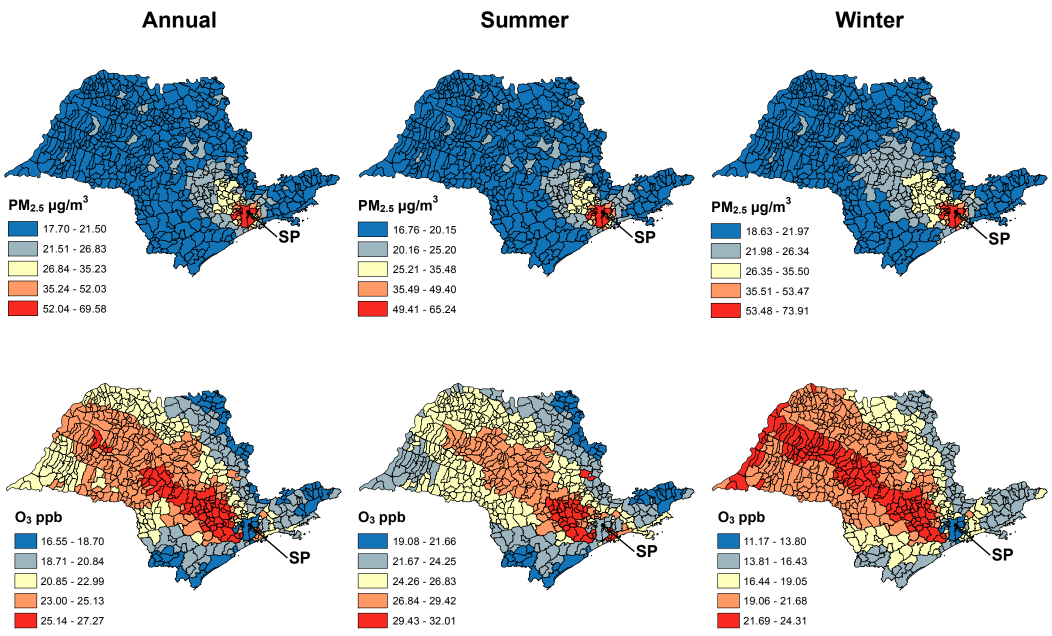

Figure S3. Fine particulate matter ($PM_{2.5}$) (annual average) and ozone (O_3) (average of 1 h maximums) in the.Fossil Fuel scenario.

Section S7: Differences in Air Pollution in Select Groups of Municipalities

Table S4. Difference in fine particulate matter ($PM_{2.5}$) and ozone concentrations between the scenarios for different municipal groupings and in the five most populous individual municipalities. Positive numbers indicate that concentrations were higher in the Ethanol Expansion compared to the Fossil Fuel scenario.

	$PM_{2.5}$ µg/m^3 (Annual)	Ozone ppb (Warm-Season)
All municipalities *	0.35 ± 1.03	1.00 ± 0.31
50 least populous *	0.01 ± 0.09	1.03 ± 0.12
50 most populous *	2.42 ± 2.37	0.44 ± 0.69
São Paulo	6.61	−0.67
Guarulhos	4.01	0.02
Campinas	1.50	0.53
São Bernardo	6.78	−1.01
Santo Andre	4.56	−0.56

* These are simple means and standard deviations (they are not population-weighted).

Section S8: Health Impacts by Cause

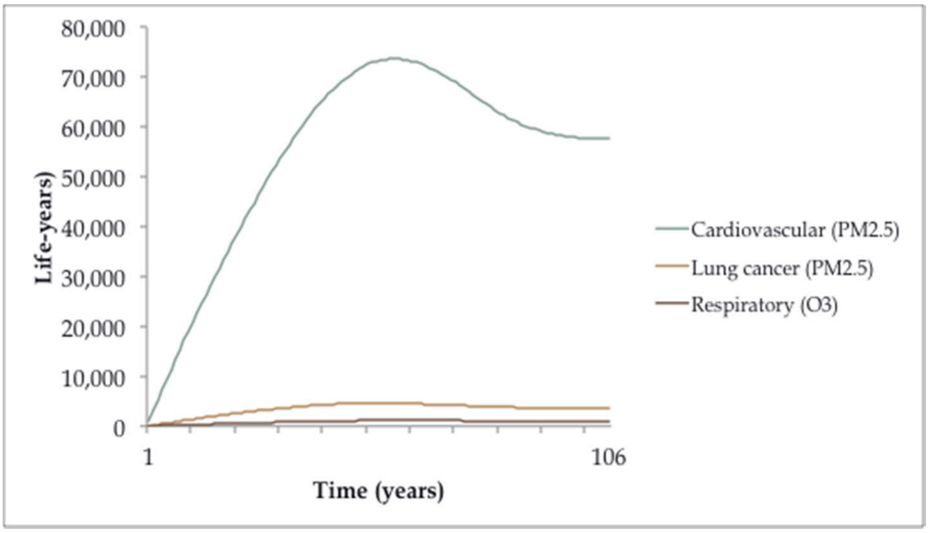

Figure S4. Cause-specific gains in life years over the full 106 follow-up period in the Fossil Fuel scenario. Graph depicts impacts using the central estimate of the concentration-response functions only.

Section S9: Impacts Using Coefficients for All-Cause Mortality ($PM_{2.5}$)

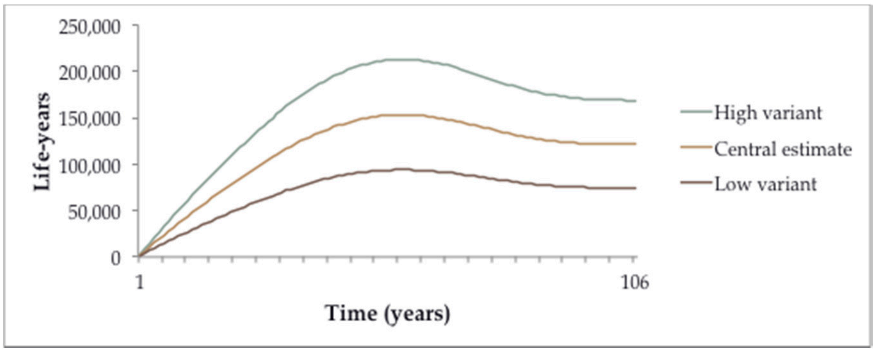

Figure S5. Gain in life years over the full 106 follow-up period in the Fossil Fuel scenario from all-cause mortality, using concentration-response coefficients from Hoek et al. [11] as updated by Forestieri et al. [12]. High and Low variant refers to the use of the 5th and 95th confidence interval of the concentration-response function, respectively.

References

1. CETESB. *Emissões Veiculares no Estado de São Paulo 2011*; CETESB: São Paulo, Brazil, 2012
2. Alonso, M. *Previsão de Tempo Químico Para a AMERICA do Sul: Impacto Das Emissões Urbanas Nas Escalas Local e Regional*; INPE: São Jose dos Campos, Brazil, 2011.
3. MCT. *Segundo Inventário Brasileiro de Emissões Antrópicas de Gases do Efeito Estufa*; Ministério da Ciência e Tecnologia: Brasília, Brazil, 2010.
4. ANFAVEA. *Brazilian Automotive Industry Yearbook*; Brazilian Automotive Industry Association: Sao Paulo, Brazil, 2010.
5. U.S. Environmental Protection Agency. Technical Support Document: Preparation of Emissions Inventories for the Version 4.1, 2005-Based Platform. Available online: https://www.epa.gov/sites/production/files/2015-11/documents/2005v4.1_main.pdf (accessed on 28 June 2016).
6. Miller, B. IOMLIFET: A Spreadsheet System for Life Table Calculation for Health Impact Assessment. Available online: http://www.iom-world.org/research/research-expertise/statistical-services/iomlifet/ (accessed on 15 February 20160).

7. Miller, B.; Hurley, J. *Comparing Estimated Risks for Air Pollution with Risks for Other Health Effects*; Institute of Occupational Medicine: Edinburgh, UK, 2006.
8. Ministério da Saúde. Mortalidade-São Paulo (2011). Available online: http://tabnet.datasus.gov.br/cgi/deftohtm.exe?sim/cnv/obt10SP.def (accessed on 21 February 2014).
9. Instituto Brasileiro de Geografia e Estatística. Censo Demográfico 2010: Tabela 1.12—População Residente, por Sexo e Grupos de Idade, Segundo as Grandes Regiões e as Unidades da Federação-2010. Available online: http://www.ibge.gov.br/home/estatistica/populacao/censo2010/tabelas_pdf/Brasil_tab_1_12.pdf. (accessed on 21 February 2014).
10. Instituto Brasileiro de Geografi a e Estatística. Censo Demográfico 2010: Tabela 1.1.1—População Residente, por Situação do Domicílio e Sexo, Segundo os Grupos de Idade-Brasil-2010. Available online: http://www.ibge.gov.br/home/estatistica/populacao/censo2010/caracteristicas_da_populacao/caracteristicas_da_populacao_tab_brasil_zip_ods.shtm. (accessed on 21 February 2014).
11. Hoek, G.; Krishnan, R.; Beleen, R.; Peters, A.; Ostro, B.; Brunekreef, B.; Kaufman, J. Long-term air pollution exposure and cardio-espiratory mortality: A review. *Environ. Health* **2013**, *12*, 43.
12. Forestiere, F.; Kan, H.; Cohen, A. Updated Exposure-Response Functions Available for Estimating Mortality Impacts. In *WHO Expert Meeting: Methods and Tools for Assessing the Health Risks of Air Pollution at Local, National and International Level*; World Health Organization Regional Office for Europe: Copenhagen, Denmark, 2014.

© 2016 by the authors. Submitted for possible open access publication under the terms and conditions of the Creative Commons Attribution (CC-BY) license (http://creativecommons.org/licenses/by/4.0/).
